# Promoter methylation-mediated repression of *UNC5* receptors and the associated clinical significance in human colorectal cancer

**DOI:** 10.1186/s13148-021-01211-5

**Published:** 2021-12-18

**Authors:** Dong Dong, Runshi Zhang, Jie Shao, Aimin Zhang, Yichao Wang, Yunli Zhou, Yueguo Li

**Affiliations:** 1 Department of Laboratory, Tianjin Medical University Cancer Institute and Hospital, Tianjin’s Clinical Research Center for Cancer, Key Laboratory of Cancer Prevention and Therapy, National Clinical Research Center for Cancer, Huanhuxi Road, Hexi District, Tianjin, 300060 People’s Republic of China; 2 Department of Clinical Laboratory, Xi’an No. 1 Hospital, Xi’an, 710002 Shaanxi People’s Republic of China; 3 Department of Clinical Laboratory Medicine, Taizhou Central Hospital (Taizhou University Hospital), No.999 Donghai Road, Jiaojiang District, Taizhou, 318000 Zhejiang Province People’s Republic of China

**Keywords:** UNC5 receptors, Methylation, Expression, Colorectal cancer, Prognosis

## Abstract

**Background:**

Deregulated methylation of tumor suppressor genes is a hallmark event in colorectal cancer (CRC) carcinogenesis. *UNC5* receptors, down-regulated in various human malignancies due to epigenetic alterations, have been proposed as putative tumor suppressor genes. In this study, we focused on the methylation-mediated inhibition of UNC5 receptors and the associated clinical significance in CRC.

**Methods:**

Methylation and expression analysis was performed in TCGA datasets. And the results were confirmed in vitro in CRC cell lines treated with 5-aza-deoxycytidine. Then, the expression and epigenetic alterations of *UNC5* receptors were evaluated in clinical specimens. Moreover, the diagnostic and prognostic values of the methylation alterations were also analyzed.

**Results:**

Methylation-mediated repression was observed in *UNC5C* and *UNC5D*, but not in *UNC5A* and *UNC5B*, which was confirmed in CRC cell lines. Except for *UNC5B*, significantly elevated methylation was observed in *UNC5A*, *UNC5C*, and *UNC5D* in CRC. The discrimination efficiency of the three receptors was comparable with that of *SEPT9.* Kaplan–Meier curve survival analysis showed that hypermethylation of *UNC5A*, *UNC5C* and *UNC5D* was associated with poor progression-free and overall survival. Moreover, methylation levels of *UNC5C* and *UNC5D* were independent predictors of CRC progression-free (*P* = 0.001, *P* = 0.003, respectively) and overall survival (*P* = 0.008, *P* = 0.004, respectively).

**Conclusions:**

Hypermethylation of *UNC5C* and *UNC5D* mediates the repression and has promising diagnostic and prognostic values in CRC.

**Supplementary Information:**

The online version contains supplementary material available at 10.1186/s13148-021-01211-5.

## Introduction

Colorectal cancer (CRC) accounts for nearly one-tenth of all cancers and is the fourth leading cause of cancer-related death worldwide [1]. Deregulated DNA methylation is one of the hallmark events in colorectal carcinogenesis, characterized by global hypomethylation of the genome and paradoxical hypermethylation of CpG islands [2]. The former is thought to influence CRC development by inducing chromosomal instability, while the latter could result in transcriptional silencing of tumor suppressor genes [2]. Hypermethylation of the CpG islands around promoters of specific suppressor genes, predominantly maintained by the DNA methyltransferase 1 (DNMT1), can be stably inherited for multiple generations in tumor cells and is involved in the process of carcinogenesis and progression of CRC [3, 4]. Besides, approaches for convenient and reliable detection of methylation with small amounts of DNA are available now [5]. These characteristics make DNA methylation as favorable CRC molecular marker with significant clinical value. Accordingly, extensive efforts have been made for comprehensive assessment of aberrantly methylated genes in CRC, which may not only improve our understanding of the epigenetic regulation of the disease but also identify CRC-related suppressor genes that might influence clinical management of patients [6].

UNC-5 family members, including four homologs (UNC5A-D), were identified as receptors of netrin-1 [7]. Due to their ability to trigger apoptosis in the absence of netrin-1, UNC5 receptors have been reported to function as dependence receptors [8]. For instance, apoptosis induced by UNC5 receptors in normal colonic epithelium is precisely regulated by varied expression of netrin-1, with highest expression in the crypts and lowest expression in the upper portion of the villi [9]. This indicated that the system may play essential roles in maintaining the normal intestinal epithelial microenvironment, and its de-regulation might promote the formation of hyperplasia, adenoma, or adenocarcinoma [10]. Therefore, UNC5 receptors have been proposed as putative tumr suppressor genes, with down-regulated expression in a variety of human malignancies due to genetic and epigenetic alterations [8]. We previously showed that loss of heterozygosis and DNA methylation contributed to the inactivation of UNC5C [11] and UNC5D [12] in renal cell carcinoma. Moreover, down-regulated expression of UNC5D in prostate cancer due to the hypermethylation of promoter was involved in the distant metastasis of the disease [13]. And increasing attention has been focused on the repression of UNC5 receptors and their biological function in human malignancies.

However, apart from UNC5C, few studies have focused on methylation-mediated inhibition of UNC5 receptors and the associated clinical significance in human colorectal cancer [14–17]. Although loss of expression of UNC5C due to epigenetic alterations has been observed in human colorectal cancer [14–17], its clinical significance in CRC diagnosis or prognosis remains to be evaluated. Considering their importance in the maintenance of intestinal epithelial homeostasis, adequate assessment of the methylation-mediated repression of UNC5 receptors in CRC may have important clinical applications. In the present study, a comprehensive assessment of methylation-mediated repression of UNC5 receptors was performed. The methylation alterations of receptors with clinical significance were assessed quantitatively, and their potential clinical values in diagnosis and prognosis of CRC were also evaluated.

## Materials and methods

### CRC clinical specimens

Tumor and corresponding non-cancerous tissues were obtained from 59 CRC patients, who underwent surgery at Tianjin Medical University Cancer Institute and Hospital between January 2016 and April 2020. All procedures performed in studies involving human participants were approved by the Research Ethics Committee of Tianjin Medical University Cancer Institute and Hospital, and in accordance with the 1964 Helsinki Declaration ethical standards, and all specimens were collected following written informed consent. Inclusion criteria for study participants including CRC as primary tumor confirmed by pathologic examination, complete follow-up of patients, and deaths caused by tumors and related complications. And participants who do not meet any of the above criteria were excluded from this study. Disease status was assessed by serial CT scans and another diagnostic testing as needed. The follow-up time ranged from 6 to 50 months, and the cutoff date follow-up was 10 April 2020.

### Cell lines and DNA methyltransferase inhibitor treatment

The human colon cancer cell lines SW480 and SW620 involved in this study were obtained from the Cell Bank of Chinese Academy of Medical Sciences (Beijing, China). For demethylation assays, cell lines were treated with 10 μM of 5-aza-2-deoxycytidine (5-Aza-dC, Sigma-Aldrich) for 3 days. DMEM medium containing 10% FBS (Gibco-BRL, Gaithersburg, MD, USA) and 1% penicillin/streptomycin, as well as 5-Aza-dC or 0.1% DMSO alone was changed every 24 h.

### Reverse transcriptase-polymerase chain reaction (RT-PCR) and real-time PCR

Quantitative real-time PCR was performed as previously described [11]. All primers used in this study are shown in Additional file 1: Table S1.

### Methylation analysis

Genomic DNA (500 ng) was bisulfite converted by EZ DNA-Methylation Gold kit [18] (Zymo Research, Irvine, CA, USA). Bisulfite genomic sequencing (BGS) analyses were conducted as described previously [19]. PCR for Methylight assays and the calculation for the percentage of methylated reference (PMR) was performed as described previously [20]. Pyrosequencing assays were performed by Saigon Biotech Co. Ltd., Shanghai (China). Sequence information for the primers and probes used is listed in Additional file 1: Table S1.

### Bio information analysis

The CpG islands around promoter were predicted by Methprimer 2.0 (http://www.urogene.org/methprimer2/) [21]. TPM expression data of CRC and paired normal tissues were downloaded from UCSC Xena hubs (http://xena.ucsc.edu/) [22], and the expression and the methylation level of genes in CRC from TCGA database (http://cancergenome.nih.gov/) [23] were analyzed using R (version 4.0.1). Whole genes methylation were generated using the Methylation Plotter web tool (http://maplab.imppc.org/methylation_plotter/) [24].

### Statistical analysis

Statistical significance was determined with the nonparametric Mann–Whitney U-test (differences between groups), paired Student's *t*-test (differences between CRC and paired noncancerous tissues). Nonparametric Spearman's correlation coefficients method was used to evaluate the association between methylation or expression levels. Receiver operating characteristic (ROC) curves were generated to assess diagnostic efficiency. Overall and progress survival was analyzed using the Kaplan–Meier product-limit method and log-rank test. All of these statistical analyses were performed with SPSS 23.0 software (SPSS Inc., Chicago, IL, USA). Values of *P* ≤ 0.05 were considered statistically significant.

## Result

### Whole gene methylation analysis of *UNC5* receptors in TCGA dataset of CRC

**Fig. 1:**
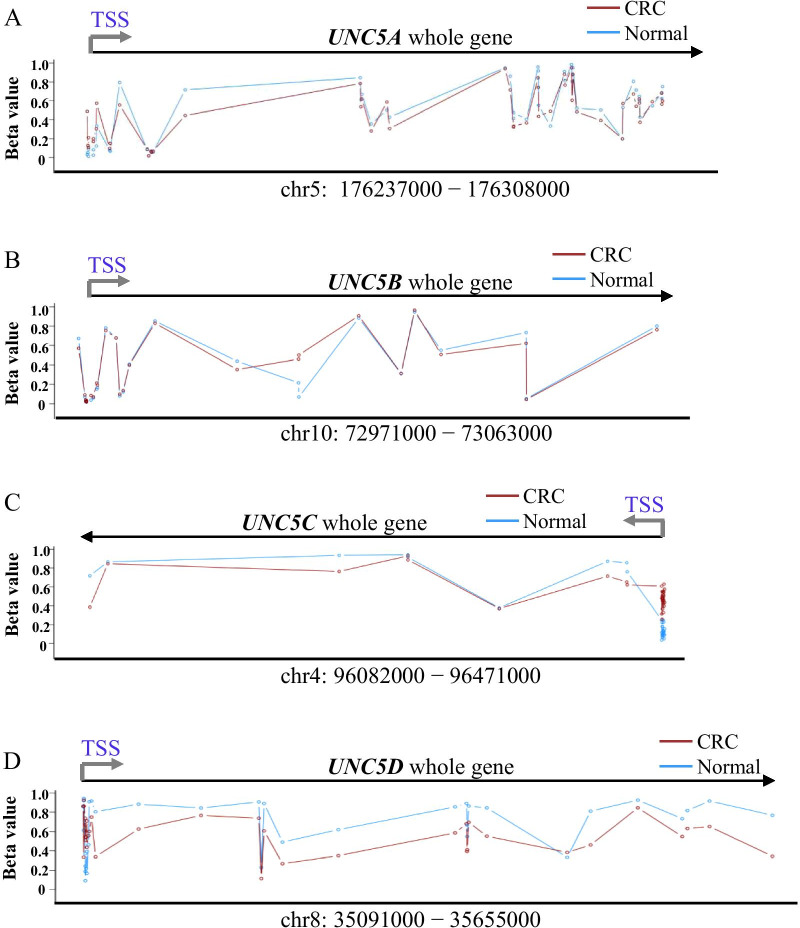
Whole gene methylation analysis of *UNC5* receptors in TCGA dataset of CRC. Whole gene methylation analysis of *UNC5* receptors in TCGA dataset of CRC using Methylation Plotter. Based on the 450 K array data in TCGA database, the mean methylation levels (beta values) of CpG sites across each gene in CRC (red) and paired normal tissues (blue) were calculated. Representation of individual receptor, **A** *UNC5A*, **B** *UNC5B*, **C** *UNC5C*, **D** *UNC5D*. Chromosome location of each receptor was as indicated. TSS, transcription start site

To gain insight into the overall methylation alterations of *UNC5* receptors in CRC, we performed whole gene methylation analysis using the web tools Methylation Plotter. Based on the 450 K array data in TCGA database, the mean methylation levels (beta values) of CpG sites across each member in CRC and paired normal tissues were calculated. As shown in Fig. 1, *UNC5A* exhibited some degree of changes, but with low methylation level around TSS (Fig. 1A). As for *UNC5B*, nearly no methylation alteration was observed, and CpG sites around TSS remained hypomethylated in both normal and tumor tissues (Fig. 1B). However, typical methylation changes of tumor suppressor genes in CRC were observed in *UNC5C* and *UNC5D*, that was, global hypomethylation of the whole gene and specific hypermethylation of CpG islands around the TSS (Fig. 1C, D). These results indicated that methylation may exert different regulatory effects in the expression of *UNC5* receptors.

### Promoter methylation and expression of *UNC5* receptors in TCGA dataset of CRC

**Fig. 2:**
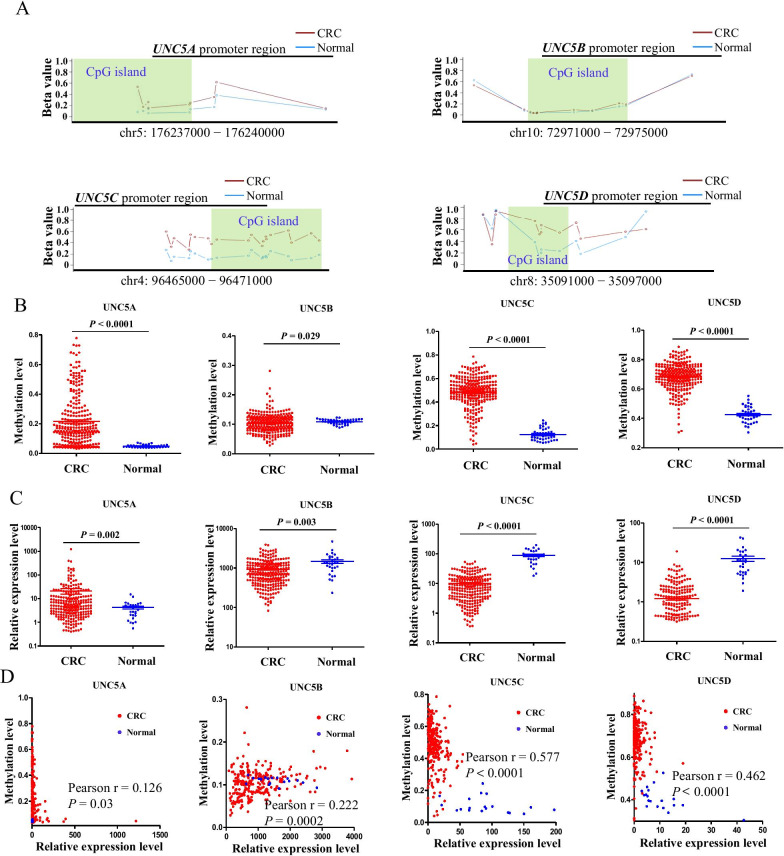
Promoter methylation and expression of *UNC5* receptors in TCGA dataset of CRC. **A** promoter methylation analysis of *UNC5* receptors in TCGA dataset of CRC using Methylation Plotter. **B** scatter plots of the mean promoter methylation levels of *UNC5* receptors in TCGA dataset of CRC. Each point represented the mean methylation levels (beta values) of promoter CpG sites in CRC (red) or paired normal tissues (blue). **C** Comparison of TPM expression of each receptor between normal and tumor tissues of CRC. **D** The association between expression and methylation of each receptor

Next, we focused our analysis on the methylation alterations in the CpG islands around the promoter regions of *UNC5* receptors. The mean beta values of corresponding CpG sites in promoter region for every patient from TCGA database were shown in Fig. 2A, B. Although the beta values of *UNC5A* were elevated in CRC tissues, the mean value in CRC tissues was only about 0.22. And the mean beta values of *UNC5B* were at a very low level, about 0.10, in both normal and tumor tissues (Fig. 2A, B). On the contrary, the methylation levels of *UNC5C* and *UNC5D* were elevated in CRC, with mean beta values of 0.48 and 0.68, respectively (Fig. 2A, B). It is widely recognized that probes with beta values below or equal to 0.2 were classified as unmethylated, between 0.2 and 0.8 as intermediate, and higher or equal to 0.8 as methylated [25, 26]. Thus, among the members of this family, promoter regions *UNC5C* and *UNC5D* were hypermethylated.

Differential expression analysis of *UNC5* receptors between CRC and paired adjacent noncancerous tissues in the TCGA database were further performed. As shown in Fig. 2C, *UNC5A* was observed with some degree of elevated expression, and expression of *UNC5B* was not apparently altered in CRC. Conversely, expression of *UNC5C* and *UNC5D* was down-regulated nearly tenfold in CRC, respectively. Further correlation analysis between promoter methylation and gene expression levels indicated that expression of *UNC5C* and *UNC5D,* but not *UNC5CA* and *UNC5B*, were highly correlated with the methylation levels (Fig. 2D). These observations with data from TCGA database implicated *UNC5C* and *UNC5D* as methylation-driven genes, with expression levels significantly affected by DNA methylation events.

### Verification of methylation-mediated repression of *UNC5* receptors in CRC cells

**Fig. 3:**
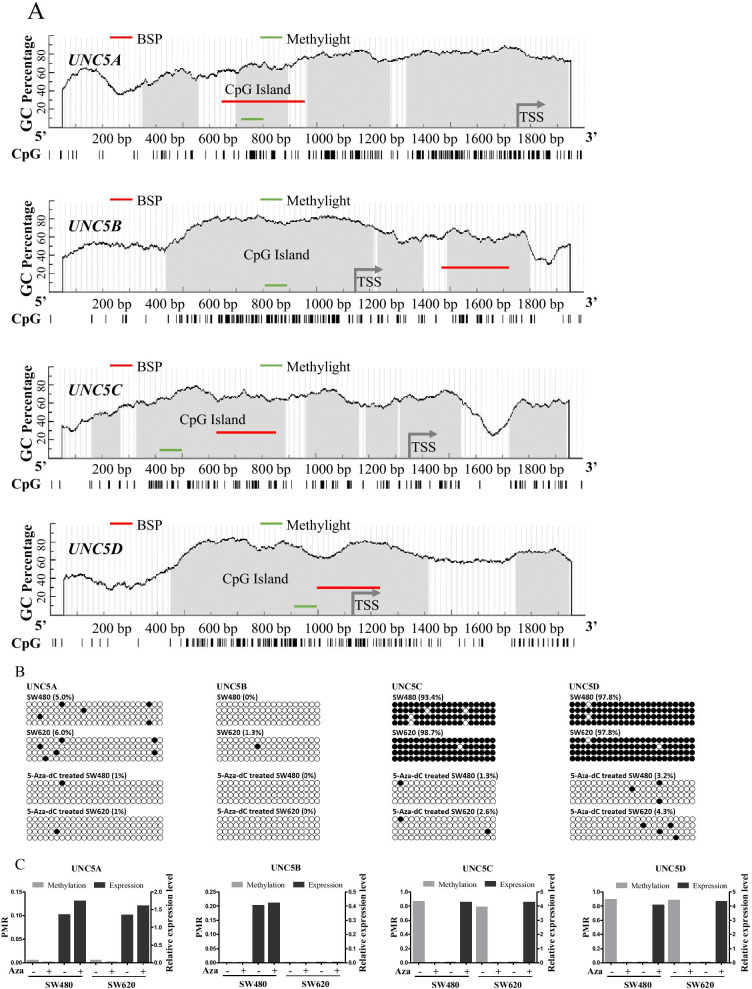
Verification of methylation-mediated repression of *UNC5* receptors in CRC cells. **A** detection methods established for methylation evaluation. CpG islands spanning the promoter region of *UNC5* receptors*.* Horizontal bars, CpG sites; detection area for BGS and methylight are indicated. TSS, transcription start site; BSP, bisulfite sequencing primers. **B** BGS results of *UNC5* promoters in CRC cell lines treated with 5-aza-2′-deoxycytidine; Circles, CpG sites analyzed; row of circles, individual promoter allele cloned and sequenced; filled circle, methylated CpG sites; open circle, unmethylated CpG site. C, methylight results of *UNC5* promoters in CRC cell lines treated with 5-aza-2′-deoxycytidine. Real-time PCR results were displayed correspondingly

Next, quantitative detection methods were established to verify the promoter methylation-mediated repression of *UNC5* receptors in CRC cell lines. As shown in Fig. 3A, bioinformatics analysis revealed that the *UNC5* receptor genes harbor typical DNA sequence fulfilling the criteria for CpG islands around the promoter region. And oligonucleotides of four receptors for BSP and methylight detection were designed accordingly. To demonstrate that CpG methylation was functionally associated with expression of *UNC5* receptors, CRC cell lines SW480 and SW620 were treated with the DNA demethylation reagent 5-azadC. The conversion effect of 5-azadC was confirmed by BGS analysis (Fig. 3B). As shown in Fig. 3C, such treatment exerted little effects on the expression of *UNC5A* and *UNC5B* but restored the expression *UNC5C* and *UNC5D*. *UNC5B*, with little alterations in methylation and expression in CRC and was left out of subsequent analyses.

### Methylation-mediated repression of *UNC5* receptors in clinical samples of CRC

**Fig. 4:**
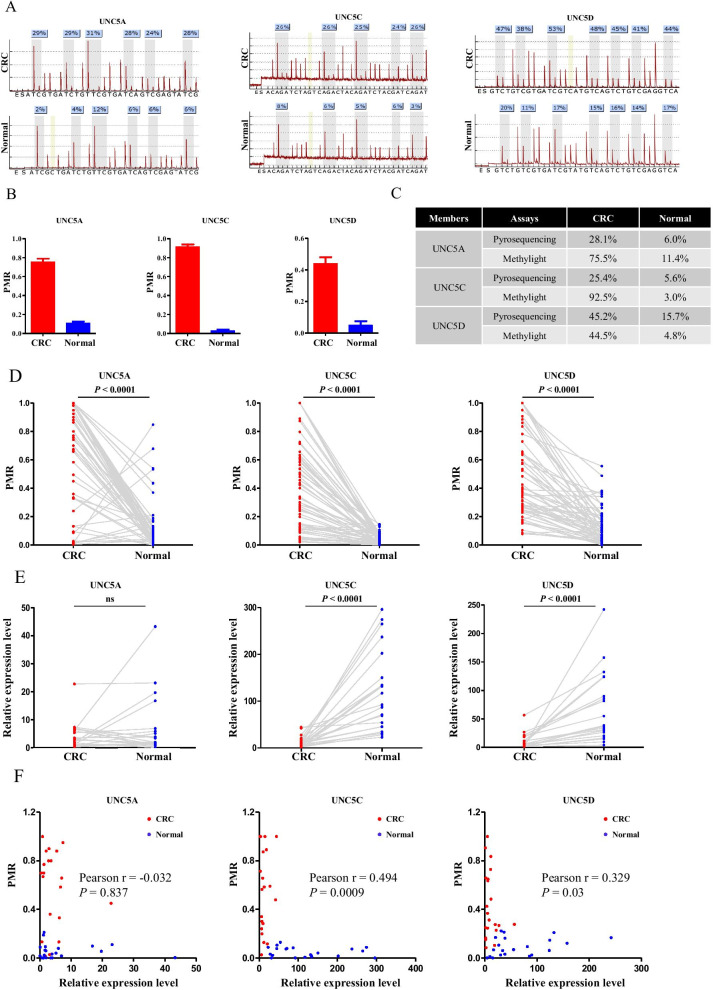
Methylation-mediated repression of UNC5 receptors in clinical samples of CRC. **A** Pyrosequencing results of *UNC5A, UNC5C* and *UNC5D* in randomly selected paired CRC specimens. **B** The corresponding methylight results of *UNC5A, UNC5C* and *UNC5D* in the selected CRC specimens. **C** Comparison of results from pyrosequencing and methylight assay. **D** Scatterplots of the methylation levels of *UNC5A, UNC5C* and *UNC5D* detected by methylight in 59 paired CRC specimens. **E** Scatterplots of the relative expression levels of *UNC5A, UNC5C* and *UNC5D* detected by real-time PCR in 21 paired CRC specimens. **F** The correlation between the expression of and the promoter methylation levels of *UNC5A, UNC5C* and *UNC5D* in 21 paired CRC specimens

The study was extended to primary tumors of CRC. To assess the performance of the methylight assay in clinical samples, pyrosequencing assay for *UNC5* receptors was also performed in randomly selected paired specimens. Elevated methylation of *UNC5A*, *UNC5C*, and *UNC5D* in CRC was confirmed by both pyrosequencing and assay methylight assays (Fig. 4A, B). Differential methylation status of the three *UNC5* receptors in paired CRC samples was verified in both assays (Fig. 4C). The results of methylight assays in 59 paired samples showed markedly increased methylation of the *UNC5* receptors in tumor in contrast to that in the adjacent non-cancerous tissues (Fig. 4D). Among those samples, mRNA of 21 pairs was available (Fig. 4E). Expression of *UNC5C* and *UNC5D*, but not *UNC5A*, were significantly associated with methylation levels of promoter (Fig. 4F), which was consistent with the above results.

### The clinical significance of methylation alterations of *UNC5* receptors in CRC

**Fig. 5:**
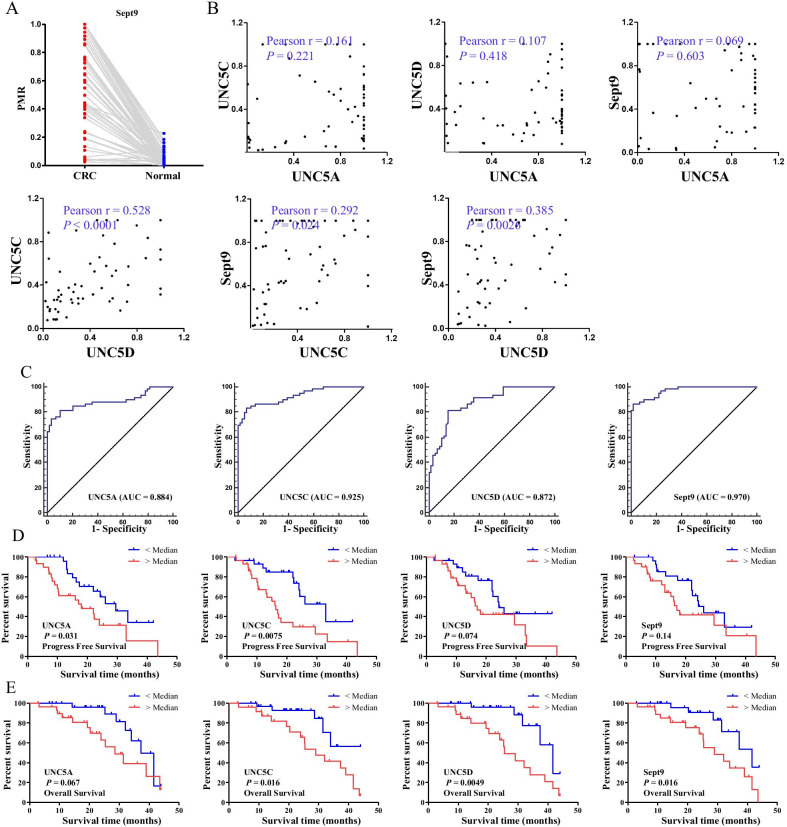
The clinical significance of methylation alterations of *UNC5* receptors in CRC. **A** scatterplots of the methylation levels of *SEPT9*, a well-known highly methylated gene in CRC tissues. **B** The correlation between the methylation levels of *UNC5A*, *UNC5C*, *UNC5D* and *SEPT9* in CRC specimens. **C** ROC analysis was performed to evaluate the performances of methylation alterations of *UNC5A*, *UNC5C*, *UNC5D* and *SEPT9* in discriminating adjacent non-cancerous tissues from CRC tissues. Kaplan–Meier analysis were performed for progression-free survival (**D**) and overall survival (**E**) in CRC patients with different methylation levels of *UNC5A*, *UNC5C*, *UNC5D* and *SEPT9*

*SEPT9* is known to be highly methylated in CRC tissues and methylated *SEPT9* in blood has diagnostic value in CRC patients [27], and was included in this study as a hyper-methylated gene control in CRC tissues. As was shown in Fig. 5A, the methylation of *SEPT9* in CRC tissues was significantly higher than the adjacent non-cancerous tissues. Moreover, methylation alterations of *UNC5C* and *UNC5D,* but not *UNC5A,* were correlated with *SEPT9* to some extent (Fig. 5B). The receiver operating curve (ROC) analysis was performed to evaluate the related discrimination efficiency. To discriminate adjacent non-cancerous tissues from CRC tissues, the performances of *UNC5A*, *UNC5C* and *UNC5D* were comparable, slightly lower than that of *SEPT9* (Fig. 5C).

Finally, we evaluated the prognostic value of methylation changes of *UNC5* receptors. Kaplan–Meier curve survival analysis showed that higher promoter methylation levels of *UNC5A* and *UNC5C* were significantly associated with poor progression-free survival (log-rank, *P* = 0.031 and = 0.0075, respectively, Fig. 5D). For *UNC5D* and *SEPT9*, a trend toward poorer progression-free survival was seen, although failed to reach statistical significance (log-rank, *P* = 0.074 and = 0.14, respectively; Fig. 5D). In addition to *SEPT9*, promoter methylation of *UNC5C* and *UNC5D* showed an association with poor overall survival (log-rank, *P* = 0.016, 0.016, and 0.0049, respectively; Fig. 5E). In Cox proportional hazards analyses of progression-free survival and overall survival, promoter methylation of *UNC5C* and *UNC5D* were associated with unfavorable patient outcomes (Table 1). And HRs did not substantially change after adjustment for TNM stage. These results indicated that the promoter methylation of *UNC5C* and *UNC5D* were independent predictors of CRC survival.

**Table 1:** Univariate and multivariate of Cox proportional hazard modeling of factors associated with survival of CRC patients

Variable	Hazard ratio	(95% CI)	*P* value
(1) Univariate analysis of overall survival			
Age	0.97	0.93–1.00	0.2
Gender (female vs. male)	0.86	0.36–2.00	0.7
TNM stage	2.26	1.23–4.17	**0.009**
PMF of *UNC5A*	1.51	0.35–6.12	0.6
PMF of *UNC5C*	5.83	1.43–23.85	**0.014**
PMF of *UNC5D*	6.10	1.48–25.04	**0.012**
PMF of *Sept9*	5.58	1.21–25.63	**0.026**
(2) Multivariate analysis of overall survival			
TNM stage^1^	3.39	1.65–6.95	**0.001**
PMF of *UNC5C*^1^	15.06	3.12–72.65	**0.001**
TNM stage^2^	2.61	1.37–4.98	**0.004**
PMF of *UNC5D*^2^	10.02	2.13–47.08	**0.003**
TNM stage^3^	2.54	1.26–5.11	**0.009**
PMF of *Sept9*^3^	5.95	1.30–27.24	**0.022**
(3) Univariate analysis of progress free survival			
Age	1.00	0.97–1.06	1.0
Gender (female vs. male)	0.94	0.46–1.92	0.9
TNM stage	1.73	1.07–2.77	**0.023**
PMF of *UNC5A*	1.81	0.62–5.43	0.3
PMF of *UNC5C*	3.44	1.12–10.56	**0.03**
PMF of *UNC5D*	4.77	1.48–15.34	**0.009**
PMF of *Sept9*	2.05	0.73–5.81	0.2
(4) Multivariate analysis of progress free survival			
TNM stage^4^	1.97	1.19–3.26	**0.008**
PMF of *UNC5C*^4^	4.81	1.50–15.38	**0.008**
TNM stage^5^	1.90	1.15–3.13	**0.012**
PMF of *UNC5D*^5^	6.17	1.81–20.99	**0.004**

*P* ≤ 0.05 is considered statistically significant and shown in bold

^1–5^ different comparison groups in Multivariate analysis of survival

## Discussion

Aberrant methylation in colorectal cancer, particularly on the CpG islands of tumor suppressor genes, is thought to be involved in disease development and progression [3, 4]. In colorectal carcinogenesis, some gene-specific hypermethylation events have been reported in precancerous lesions from CRC patients [28]. Moreover, the suppressive effect of aberrant DNA methylation on suppressor genes involved in adenoma to cancer progression has also been demonstrated [6]. These findings suggest that methylation alterations may be promising biomarkers in the etiological study of colorectal cancer. A better understanding of when these epigenetic changes occur and how they are involved in colorectal progression may be of great value in the risk assessment and clinical management of this disease. In this study, we focused on UNC5 receptors, which play vital roles in the maintenance of normal intestinal epithelial microenvironmental homeostasis.

Netrin-1-UNC5 receptors signaling axis has been reported to play a key role in neuronal navigation during the development of nervous system [29]. Recently, it has also been shown to regulate diverse processes in a number of non-neuronal tissues, especially in the regulation of tumorigenesis [29]. Due to remarkable structural homology and functional similarities, coupled with the down-regulated expression in various human malignancies, the boundaries between UNC5 receptors appear to be blurred as if they share exactly the same tumor biological significance. However, this proved not to be the case. In the present study, a comprehensive assessment of methylation-mediated repression of UNC5 receptors was performed in CRC. Significant variations were observed among the four receptors. Significantly elevated methylation in CRC was observed in *UNC5A*, *UNC5C*, and *UNC5D*, but not in *UNC5B*. *UNC5B* was hypomethylated in both CRC and adjacent normal tissues, which was consistent with the previous study [30]. Moreover, although with elevated methylation in CRC tissues, methylation-mediated inhibition did not occur in *UNC5A*, but only in *UNC5C* and *UNC5D*.

Numerous studies have provided ample evidence that *UNC5C*, a tumor suppressor, is down-regulated in a large fraction of colorectal malignancies, primarily through promoter methylation [14–17]. Recently, TCGA datasets analysis involved methylation of *UNC5C* has been performed in two studies [31, 32]. However, neither of them validated the methylation alterations of *UNC5C* by cost-effective quantitative approaches with possible clinical translation values. In contrast, quantitative results from the current study with our own specimen cohort indicated that hypermethylation of *UNC5C* was an independent predictor of CRC survival. Different from *UNC5C*, *UNC5D* is the most recently identified member of UNC5 family [33]. Consistent with other members of this family, *UNC5D* has also been characterized as a tumor suppressor gene for several cancers, such as neuroblastoma [34, 35], renal cell carcinoma [12], prostate cancer [13], and bladder cancer [36]. Recently, Uhan et al. evaluated the methylation status of probe cg13561879 located in the promoter region of *UNC5D* by methylation-specific high-resolution melting analysis (MS-HRM), and found that hypermethylation of this probe could be served as a potential novel diagnostic biomarker for colorectal cancer, but with no significant prognostic value [37]. Methylight, the quantitative method adopted in this study, relies on methylation-specific primers and probes [38]. As for *UNC5D*, 9 CpG sites in total were overlapped by the probe (4 sites) and primers (5 sites). In contrast, because more sites are covered, the methylight assay can reflect the methylation status of CpG islands much more accurately than the MS-HRM assay, which may partially explain the difference in Cox proportional hazards regression results for overall survival.

Some limitations of this study should also be noted. The conclusion that hypermethylation of *UNC5C* and *UNC5D* has diagnostic and prognostic values in CRC was drawn from a relatively small CRC cohort. A large cohort should be used to verify the clinical significance of methylated *UNC5* receptors for patients with CRC. Another limitation of this study is the lack of control group with benign colorectal polyps or adenomas. Implementation of this work with an enriched control group will further elucidate the applicability of the methylation assay for *UNC5* receptors. In addition, recent work has provided ample evidence that genome-wide DNA methylation is altered in aging and age-related diseases [39]. However, due to the limited number of cases included in this study, the potential age-related differences in the methylation of *UNC5* receptors were not considered. Finally, *UNC5C*, *UNC5D*, and *SEPT9* were observed with similar methylation patterns in CRC, which suggests that elevated methylation of *UNC5C* and *UNC5D* may have the same clinical applications as *SEPT9*. As is known that the methylated *SEPT9* (mSEPT9) assay was the first blood-based methylation test approved by the United States Food and Drug Administration for colorectal screening. Quantitative mSEPT9 levels have been successfully applied for the screening, diagnosis, monitoring, and prognosis of CRC [40–42]. The potential of methylated *UNC5C* or *UNC5D* in circulating tumor DNA as biomarker for CRC should also be taken into consideration in future study.

## Conclusions

In summary, this is the first study that comprehensively evaluates the aberrantly methylation of *UNC5* receptors and the associated clinical significance in human colorectal cancer. Except for *UNC5B*, *UNC5A*, *UNC5C* and *UNC5D* were observed with significantly increased methylation levels in the promoter region. Methylation-mediated repression was observed in *UNC5C* and *UNC5D*, but not in *UNC5A* and *UNC5B* in CRC. A cost-effective and PCR-based quantitative DNA methylation detection approach with high sensitivity, methylight, was employed for quantitative methylation evaluation. And it was explored that hypermethylation of *UNC5A*, *UNC5C*, and *UNC5D* was associated with poor progression-free and overall survival. And methylation levels of *UNC5C* and *UNC5D* were independent predictors of CRC survival. Our findings proposed that hypermethylation of *UNC5* receptors could be potential diagnostic and prognostic biomarkers for CRC.

## Supplementary Information

**Additional file 1.** Sequence information for the primers and probes.

## Data Availability

The datasets analyzed in the current study are available from the corresponding author on reasonable request.
