# Supplementary material for: Promoter methylation-mediated repression of UNC5 receptors and the associated clinical significance in human colorectal cancer
Source: Clin Epigenetics. 2021 Dec 18;13:225. doi: 10.1186/s13148-021-01211-5 (PMC8684698; doi:10.1186/s13148-021-01211-5)
Supplement: Supplementary file 1 — Additional file 1. Sequence information for the primers and probes. [file 13148_2021_1211_MOESM1_ESM.docx]

**Table S1.** Sequence information for the primers and probes.

| Application | Annotation | Primer Sequence |
| --- | --- | --- |
| Real-time PCR  Sequencing(BGS)  Methylight PCR | UNC5A  UNC5C  UNC5D  GAPDH  UNC5A  UNC5B  UNC5C  UNC5D  UNC5A-primer  UNC5A-probe  UNC5B-primer  UNC5B-probe  UNC5C-primer  UNC5C-probe  UNC5D-primer  UNC5D-probe  Sept9-primer  Sept9-blocker  Sept9-probe  Alu4-primer  Alu4-probe | 5’- GACACCCGCAACTGTACCA -3’  5’- CCTTCTTCCGGCAATAAACGA -3’  5’- AAGTGAGCATTGAGATTTCGCGCC -3’  5’- AGATATGCAATGCGCACATACGCC -3’  5’- GAGAGTGAACGAGAAACC -3’  5’- GTCTGATGGAGTAGGGAA -3’  5’- ACCCACTCCTCCACCTTTGA -3’  5’- CTGTTGCTGTAGCCAAATTCGT -3’  5’- AAAATAACTCTACTCCACAATCACT -3’  5’- TTAAGGTTATAGAGTTGGAAGGTGAAG -3’  5’- CACCAAAACTAACAAACCAAACTC -3’  5’- GTGTAGGAGTTAGGGTTTGAGTTTT -3’  5’- CTTCCTATATTCAACAAATCTTTTC -3’  5’- GTAAATTTATAGTTGGTTTTTTGGG -3’  5’- TAATCTCCAATCTCAATATTCTCAC -3’  5’- AGTAGAGTTATTTTTTGAAGATTTT -3’  5’- AAAAACACAACCCCAACCAC -3’  5’- CGTTTTCGGGGTTTAAATATCGC -3’  5’- AATTTCCCTAACCCCTCGAC -3’  5’-FAM- CTTCAAACGCAACACACCCGACT -BHQ1-3’  5’- TCGCGTAATTTCGGAGGT -3’  5’- GCCCCTCTAACTCCGAAT -3’  5’-FAM- CTCGCCTAACTCCGACGCTAT -BHQ1-3’  5’- AGTTCGTATCGGTTTCGTTAATCG -3’  5’- CCCGAACCACTAAACTATATCCAAT -3’  5’-FAM- AACTCAACCTACCGCCTACCACCTTCT -BHQ1-3’  5’- TTCGGAGCGTGAAGAAGAGTCG -3’  5’- CGTTGGTTTTCGTGGTTGGGG -3’  5’-FAM-CCTCCGCCGCCGCCTACGATAAC-BHQ1-3’  5’- GattYGtTGtttAttAGttATtATGT -3’  5’- Gatt-dS-GtTGtttAttAGttATtATGT -3’  5’- gttattatgttggattttgtggttaatgtgtag-C3 -3’  5’-FAM- ttaaccgcgaaatccgac -BHQ1-3’  5’-GGTTAGGTATAGTGGTTTATATTTGTAATTTTAGTA-3’  5’-ATTAACTAAACTAATCTTAAACTCCTAACCTCA-3’  5’-FAM-CCTACCTTAACCTCCC-MGB-3’ |
